# Phosphorylated and Non-phosphorylated Leucine Rich Amelogenin Peptide Differentially Affect Ameloblast Mineralization

**DOI:** 10.3389/fphys.2018.00055

**Published:** 2018-02-08

**Authors:** Elvire Le Norcy, Julie Lesieur, Jeremy Sadoine, Gaël Y. Rochefort, Catherine Chaussain, Anne Poliard

**Affiliations:** ^1^EA2496 Faculté de Chirurgie Dentaire, Université Paris Descartes USPC, Paris, France; ^2^APHP, Hôpital Bretonneau, Service d'Odontologie, Paris, France

**Keywords:** amelogenin, leucine-rich amelogenin peptide, LRAP, phosphorylation, hydroxyapatite, ameloblasts, ameloblastic cell line, tooth germ

## Abstract

The Leucine Rich Amelogenin Peptide (LRAP) is a product of alternative splicing of the *amelogenin* gene. As full length amelogenin, LRAP has been shown, in precipitation experiments, to regulate hydroxyapatite (HAP) crystal formation depending on its phosphorylation status. However, very few studies have questioned the impact of its phosphorylation status on enamel mineralization in biological models. Therefore, we have analyzed the effect of phosphorylated (+P) or non-phosphorylated (−P) LRAP on enamel formation in ameloblast-like cell lines and *ex vivo* cultures of murine postnatal day 1 molar germs. To this end, the mineral formed was analyzed by micro-computed tomography, Field Emission Scanning Electron Microscopy, Transmission Electron Microscopy, Selected Area Electon Diffraction imaging. *Amelogenin* gene transcription was evaluated by qPCR analysis. Our data show that, in both cells and germ cultures, LRAP is able to induce an up-regulation of *amelogenin* transcription independently of its phosphorylation status. Mineral formation is promoted by LRAP(+P) in all models, while LRAP(–P) essentially affects HAP crystal formation through an increase in crystal length and organization in ameloblast-like cells. Altogether, these data suggest a differential effect of LRAP depending on its phosphorylation status and on the ameloblast stage at the time of treatment. Therefore, LRAP isoforms can be envisioned as potential candidates for treatment of enamel lesions or defects and their action should be further evaluated in pathological models.

## Introduction

Dental Enamel is the outermost layer of the teeth and the most mineralized structure in the vertebrates, since it is constituted of at least 95% minerals. Its microstructure is composed of nanorod-like hydroxyapatite (HA) crystals arranged in a highly organized unit called the enamel prism or rod. Prism high organization leads to enamel robust mechanical properties for tissue protection against cariogenic bacteria and mechanical force upon tooth function. Enamel is formed through synthesis, growth, and organization of these rods by specialized cells, the ameloblasts, throughout the process of amelogenesis. In contrast to bone or dentin, it is acellular in its mature form. Indeed, ameloblasts are once and for all, degraded during the process of tooth eruption and consequently, they cannot regenerate and actively repair by themselves. In view of the high prevalence of dental caries and enamel defects, enamel regeneration, and repair has become a target for developing biomimetic therapeutic approaches (Cao et al., 2014; Ruan and Moradian-Oldak, 2015; Snead, 2015).

The biological processes involved in enamel formation are well characterized (Li et al., 2006). During amelogenesis, ameloblasts undergo a maturation process with a change in appearance from early, elongated secretory cells actively involved in organic extracellular matrix synthesis, to more round, mature cells involved in the degradation of this matrix and deposition of the mineral. Ameloblast extracellular matrix is known to be key for controlling growth and organization of enamel crystals during mineralization (Robinson et al., 1989; Iijima and Moradian-Oldak, 2004). It is essentially synthesized by the secretory stage ameloblasts and is composed of various structural proteins such as amelogenin, ameloblastin, enamelin, and MMP20. Among these proteins, amelogenins are the most abundant (Fincham et al., 1999; Moradian-Oldak, 2012). Native amelogenin, has been shown, in porcine teeth, to be synthesized mostly under a form phosphorylated on the single Serine-16 site. Phosphorylation affects amelogenin function since phosphorylated native porcine amelogenin (P173) inhibits calcium phosphate crystallization and stabilizes amorphous calcium phosphate while its recombinant un-phosphorylated counterpart guides the formation and organization of aligned enamel crystals (Beniash et al., 2005; Wang et al., 2007; Kwak et al., 2009; Wiedemann-Bidlack et al., 2011; Margolis et al., 2014). Different isoforms of amelogenin, mostly resulting from alternative splicing, have been evidenced in bovine and rodent enamel (Shimokawa et al., 1989; Lau et al., 1992). They are translated into amelogenin proteins that vary in length and relative abundance (Bartlett et al., 2006; Yamakoshi, 2011). Among these alternative isoforms, the Leucine Rich Amelogenin Peptide (LRAP) is the second most abundant amelogenin protein (Shimokawa et al., 1989). LRAP was observed in secretory stage ameloblasts (Iacob and Veis, 2008) and shown to be produced throughout amelogenesis (Yuan et al., 1996; Veis et al., 2000). It is a short peptide (56–59 amino acids, depending on the species) identical to the full-length amelogenin except for the majority of the exon-6 coded region that is lacking (Bonass et al., 1994). It contains the two self-assembly domains of the full-length amelogenin form (Paine and Snead, 1997; Pugach et al., 2010) and has been evidenced in mouse, porcine, bovine, and human (Goldberg, 2010). LRAP has been demonstrated to display both signaling and structural properties on dental cells. It is able to promote ameloblast or odontoblast *in vitro* differentiation (Tompkins and Veis, 2002; Sarkar et al., 2014) and can affect *in vitro* calcium phosphate formation in a very similar fashion to the full-length amelogenin (Beniash et al., 2005; Kwak et al., 2009, 2014; Wiedemann-Bidlack et al., 2011). Remarkably, the phosphorylated form of the peptide on serine 16 [LRAP(+P)] stabilized amorphous calcium phosphate (ACP) whereas the non-phosphorylated form [LRAP(–P)] was shown to guide the formation of bundles of well-aligned needle-like apatitic crystals (Le Norcy et al., 2011b). LRAP(–P) has also been recently shown to act as a surface treatment agent to enhance remineralization of altered enamel (Shafiei et al., 2015) and guide the regeneration of acid-etched enamel structure (Kwak et al., 2017).

Despite these recent observations, few studies have addressed the direct role of LRAP, on enamel mineralization in biological models, in relation to its phosphorylation status. Namely, nothing is still known on the ratio of un-phosphorylated to phosphorylated LRAP forms and whether this ratio changes during tooth development and maturation. Indeed, up to now, most researches have been performed with a recombinant non-phosphorylated LRAP peptide although amelogenins are detected *in vivo* under their phosphorylated form (Fincham and Moradian-Oldak, 1993). In a context of future therapeutic applications, the aim of this work was therefore to determine whether the phosphorylation status of LRAP impacts the nature of the mineral formed in biological systems as it does *in vitro*. To this end, the effect of the LRAP (+P) or (–P) on mineral formation was analyzed in two ameloblast-like cellular models mimicking secretory (LS8) and maturation (ALC) stage ameloblasts and in a model of *ex vivo* tooth germ culture (Chen et al., 1992; Nakata et al., 2003; Sarkar et al., 2014).

## Materials and methods

### Preparation of LRAP

Variations of the porcine LRAP (MPLPPHPGHPGYINF**S**^P^YEVLTPLKWYQNMIR HPSLLDLPLEAWPATDKTKREEVD) with and without the phosphate group on Serine-16, were synthesized commercially (NEO Peptide, Cambridge, MA, USA) and re-purified, as previously described (Nagano et al., 2009). Lyophilized peptides were weighed and dissolved in distilled de-ionized water at room temperature to yield a stock solution of 2 mg/mL. Complete solubilization of both peptides in water was verified by dynamic light scattering analyses. LRAP concentrations were confirmed by nanodrop analyses at 280 nm.

### Cell culture

The mouse ameloblastic cell lines LS8 (Chen et al., 1992) and ALC (Nakata et al., 2003) were routinely cultured in Dulbecco's Modified Eagle Medium (DMEM) supplemented with 10% Fetal Bovine Serum (FBS) and 1% Penicillin-Streptomycin (PS). ALC cultures also contained 10 ng/mL mouse Epidermal Growth Factor (mEGF) (Nakata et al., 2003). Control mouse embryonic fibroblast cells (NIH3T3) were cultured in DMEM High Glucose (DMEM HG) supplemented with 10% FBS and 1% PS. For mineralization studies, the cells were then cultured in DMEM (or DMEM HG for NIH3T3 cells) supplemented with 1% FBS, 1% PS, 50 μg/mL ascorbic acid, 5 mM β-glycerophosphate referred as the mineralizing medium. ALC cells were also supplemented with 10 ng/mL mEGF and 10^−8^M Dexamethasone. One μg/mL LRAP(+P) or LRAP(–P) were added to the mineralization medium. Controls were cultured in the same medium without LRAP peptides. Mineralization was evaluated by alizarin red staining. Cells were rinsed with Phosphate-Buffered Saline (PBS), stained with Alizarin Red Stain (2%) for 2 min then rinsed two times with PBS.

### Molar germ culture

First mandibular and maxillary molar germs (*n* = 125) were extracted from post-natal day 1 Swiss Webster mice (PND1) after euthanasia. This procedure was carried out in accordance with the French regulations on animal testing (Decree n^r^ 2013-118 of February 1st 2013 on animal protection used for scientific purposes NOR: AGRG1231951D). Germs were cultured in a mineralizing medium composed of Minimum Essential Medium α (MEM α) supplemented with 10% FBS, 0.18 mg/mL ascorbic acid, 1x Glutamine, 1% Penicillin/streptomycin, and 5 mM β-glycerophosphate. A quantity equivalent to one third of the medium volume of agar was added to each well. Thirty three ng/mL LRAP(+P) or of LRAP(–P) were added to the medium before agar addition (Tompkins et al., 2005). Five mandibular and five maxillary molar germs were separately cultured for 9 days (D9) under each condition and time point and experiments were repeated 4 times (*n* = 4). Germs were fixed on the day of extraction (D0) or after 9 days of culture, by immersion in 4% paraformaldehyde (PFA) for 30 min then rinsed with PBS and stored in 70% ethanol.

### RNA extraction and quantitative PCR

Total RNAs were extracted from cells at D0, D2, and D7, and also at D14 for ALC cells and from tooth germs at D0 and D9 using respectively RNeasy Mini Kits for the cells and RNeasy Micro Kits (Qiagen) for the molars. Five hundred and fifty nanograms of total RNA were respectively reverse transcribed to first strand cDNA using a Verso cDNA Synthesis Kit (Thermo Fisher Scientific). For quantitative PCR, mouse specific primers for *Amelx* (F: GATGGCTGCACCACCAAATC, R: CTGAAGGGTGTGACTCGGG), Actin (F: GTGGCATCCATGAAACTACAT, R: GGCATAGAGGTCTTTACGG), GAPDH (F: TGTGTCCGTCGTGGATCTGA, R: TTGCTGTTGAAGTCGCAGGAG) were used. PCR was accomplished in a Lightcycler thermocycler 480R with SYBR® Green Supermix (Bio-Rad) according to the manufacturer's instructions. Values were calculated with the LightCycler® 480 software 1.5.0 (Roche, Applied Science). Results were analyzed by the method of ΔΔCt. All data points were normalized to Actin and/or GAPDH and all samples were run in triplicate. Statistical analyses were conducted with Microsoft Excel 2011 software (Microsoft, Redmond WA, USA). A two-tailed unpaired Student T comparison test was performed (α = 0.05, ^*^p < 0.05; ^***^p < 10^−4^). LRAP(–P) and LRAP(+P) treated samples were compared to the control.

### Micro-computed tomography (Micro-CT) imaging and analyses

Germ mineralization was quantified by X-ray Micro Computed Tomography imaging (Micro-CT, Quantum FX Caliper, Life Sciences, Perkin Elmer, Waltham, MA, USA) at 90 kV and 160 μA. Tridimensional images were acquired with an isotropic voxel size of 20 μm and a rotation step of 0.1° (scan time = 3 min). Before each micro-CT acquisition, the lead citrate calibrator was scanned with an HAP phantom to assign an HAP value for each gray level of lead citrate solutions. Reconstructed files were converted into eight-bit images with fixed lower and upper brightness limits using the “CT analyzer” software (Skyscan, release 1.15.4.0, Kontich, Belgium). A binary segmentation process was applied uniformly on each data stack to separate the mineralized and non-mineralized material inside the whole germ volume. The threshold value used for binarization was manually set so that every voxel with an equal or higher value was represented as solid material, and lower values represented as space. Similar gray level values for global germ density and mineral density were set and used for analysis in all samples. In the quantifications, the mineral density corresponded to a mean of the total germ mineral content (addition of dentin and enamel) whereas the enamel volume is reflected by the ratio between the volume occupied by the enamel layer and the whole germ volume.

### Transmission electron microscope (TEM) analyses

Ten microliters of aliquots were taken from scraped regions of alizarin red stained cell cultures observed under the light microscope and placed on carbon-coated Cu grids (Electron Microscopy Sciences, Hatfield, PA, USA). Duplicate grids were prepared from a minimum of three different experiments. Images were obtained in bright field and Selected Area Electron Diffraction (SAED) modes with a Tecnai 12BT Transmission Electron Microscope (TEM) at 80 kV.

### Field emission—scanning electron microscopy (FE-SEM) analyses

PFA fixed germs were analyzed using a Field Emission—Scanning Electron Microscope (Zeiss SUPRA 40). They were air dried and placed on an SEM holder without any preparation. Lateral faces of molar cuspids were observed. Acquisitions were made using the Everhart-Thornley type Secondary Electron detector (SE2) for the first three magnifications (177–226x, 10 kx, 20 kx) and using the In-lens detector for the largest magnification (40 kx).

## Results

### Effect of LRAP phosphorylation status on ameloblast cell line mineralization

To analyze the effect of LRAP and its phosphorylation status on ameloblast mineralization, we used two murine ameloblast-like cell lines (LS8 and ALC) mimicking different stages of enamel formation as well as control murine embryonic fibroblast NIH3T3 cells. LS8 cells appear to correspond to secretory stage ameloblasts characterized by high expression of *Amelx, Ambn, Enam*, and *Mmp20* transcripts while ALC cells behave as maturation stage ameloblasts with high expression of *Odam* and *Klk4* transcripts (Sarkar et al., 2014).

Culture in mineralizing medium promoted the formation of macroscopically visible mineralization nodules after alizarin red staining, at day 7 in the LS8 cells but only very scarce and light nodules in the ALC cell cultures. After 2 weeks, ALC cells exhibited small squared mineralization nodules while the LS8 cells started to degenerate (data not shown). In contrast, control culture of NIH3T3 cells in the same medium did not lead to any mineralization even after 3 weeks of culture (data not shown). Therefore, both ameloblastic cell lines were able to mineralize but with a different kinetics (Supplementary material).

**Figure 1:**
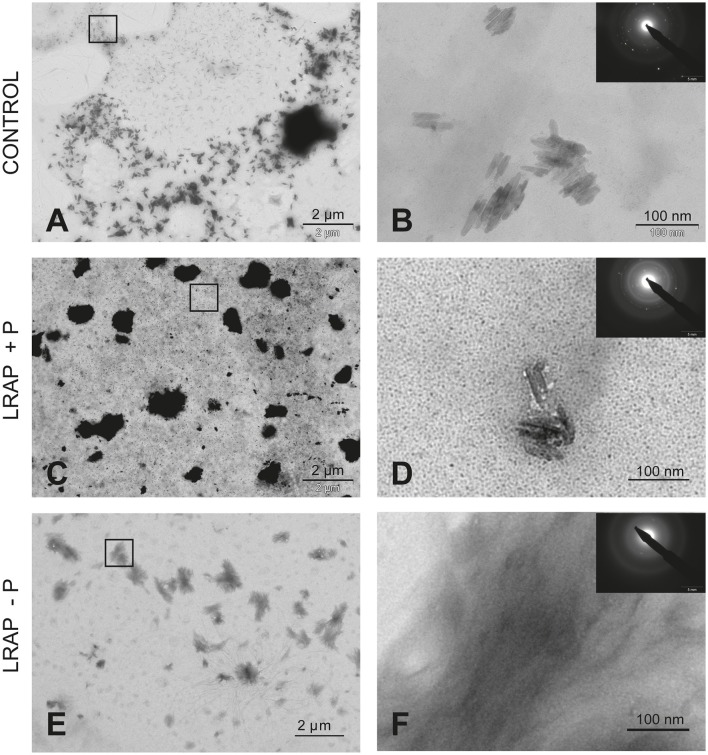
TEM and SAED analyses of mineral phases formed under mineralizing conditions by the LS8 cells in the absence and presence of added peptide. **(A,B)** CONTROL, no added peptide; **(C,D)** LRAP(+P); and **(E,F)** LRAP(–P). In **(A,C,E)**, general crystal distribution can be observed, crystal characterization is presented in **(B,D,F)**. As shown, ordered needle like HAP (based on the observed selected area diffraction patterns) crystals were formed in the control **(A,B)** and in the presence of LRAP(+P) **(C,D)** in the LS8 cells. In the presence of LRAP(–P), the HAP crystals (SAED pattern in inset) appeared thinner, longer, and grouped in bundles **(E,F)**.

**Figure 2:**
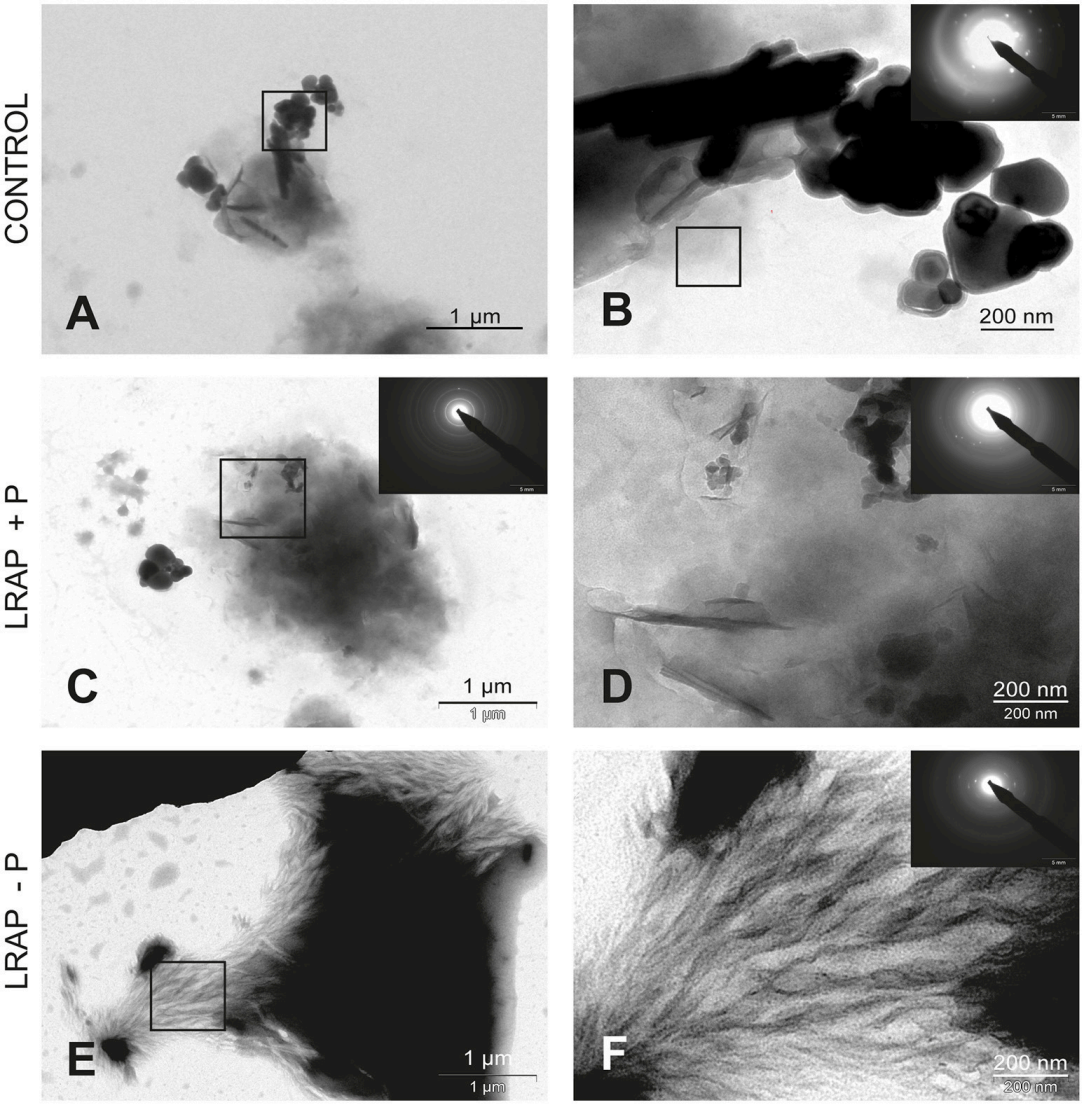
TEM and SAED analyses of mineral phases formed under mineralizing conditions by the ALC cells in the absence and presence of added peptide. **(A,B)** CONTROL, no added peptide; **(C,D)** LRAP(+P); and **(E,F)** LRAP(–P). In **(A,C,E)**, general crystal distribution can be observed, crystal characterization is presented in **(B–D,F)**. Very large round and elongated HAP crystals are present in the control **(A,B)**. Upon addition of LRAP(+P) **(C,D)** and LRAP(–P) **(E,F)**, needle-like HAP crystals were formed with LRAP(–P) potentiating, bundle formation.

To characterize the structure of the mineral formed in the various conditions, SAED analyses were performed. They showed that the mineral formed under all conditions was HAP (Figures 1, 2). Furthermore, TEM observations revealed that the mineral formed by untreated LS8 cells was composed of dispersed needle shaped HAP crystals (mean length of 43.9 ± 7.8 nm; *n* = 24) (Figures 1A,B). Upon LRAP(+P) addition, similarly dispersed but slightly longer needle shaped HAP crystals, (mean length of 56.7 ± 9.2 nm; *n* = 35) were observed (Figures 1C,D) while, bundles of fine elongated HAP crystals (mean length of 103 ± 17.8 nm; *n* = 34) were formed with LRAP(–P) (Figures 1E,F). Crystal length to width ratio (L/W) was similar in the control and LRAP(+P) treated cells (4.44 ± 0.89 and 4.4 ± 0.73, respectively) whereas it was increased in the presence of LRAP(–P) (7.38 ± 1.26; Table 1).

**Table 1:** Mean crystal length and length to width ratio formed by the LS8 and ALC cells.

	**LS8**		**ALC**	
	**Mean crystal length (nm)**	**L/W ratio**	**Mean crystal length (nm)**	**L/W ratio**
Control	43.9 ± 7.8	4.44 ± 0.89	74.9 ± 40.9	9.08 ± 3.46
LRAP(+P)	56.7 ± 9.2^*^	4.4 ± 0.73	84 ± 33.1	9.13 ± 2.97
LRAP(–P)	103 ± 17.8^***^	7.38 ± 1.26	76.2 ± 57.1	9.15 ± 2.89

Mean length of HAP crystals and length to width ratio were measured in TEM images in the control, LRAP(+P) and LRAP(-P) treated cells at D7 for LS8 and D14 for ALC. A statistically significant increase in crystal length was observed under both treatment conditions in the LS8 cells (

* p < 0.05 and

*** *p < 10^–6^). For the ALC cells, due to the large heterogeneity in crystals observed, statistical analyses of crystal length were not relevant. Similar length to width ratio were observed in the control and the LRAP(+P) treated cells with both cell lines; an increase in the ratio was observed when LS8 cells were treated with LRAP(-P)*.

In untreated ALC cells (Figures 2A,B and Table 1), a mixture of large round mineral particles and few very large elongated HAP crystals (mean length of 342.7 ± 49.5 nm) were predominantly observed with TEM and characterized by SAED although a small quantity of shorter needle-shaped HAP crystals was also present (mean length of 74.9 ± 40.9 nm; *n* = 16). On the whole, mineral structures were much larger than those found in the LS8 control cells. Upon LRAP(+P) treatment, only needle shaped HAP crystals (mean length of 84 ± 33.1 nm; *n* = 34) were observed (Figures 2C,D and Table 1). LRAP(–P) treatment promoted the formation of bundles of elongated HAP crystals (mean length of 76.2 ± 57.1 nm; *n* = 42) similar to those formed by the LS8 cell (Figures 2E,F and Table 1). While length to width ratios were similar in untreated, and LRAP(+P) or LRAP(–P) treated cells (9.08 ± 3.46, 9.13 ± 2.97, and 9.15 ± 2.89, respectively; Table 1), the mineral organization was very different between LRAP-treated and untreated cultures: large crystals were predominant in controls but absent in the peptide-treated cultures. In addition, needle-shaped crystallites were organized in bundles whereas they were randomly dispersed in the untreated cultures. This organization was more particularly evident after LRAP(-P) treatment (Figures 2E,F).

**Figure 3:**
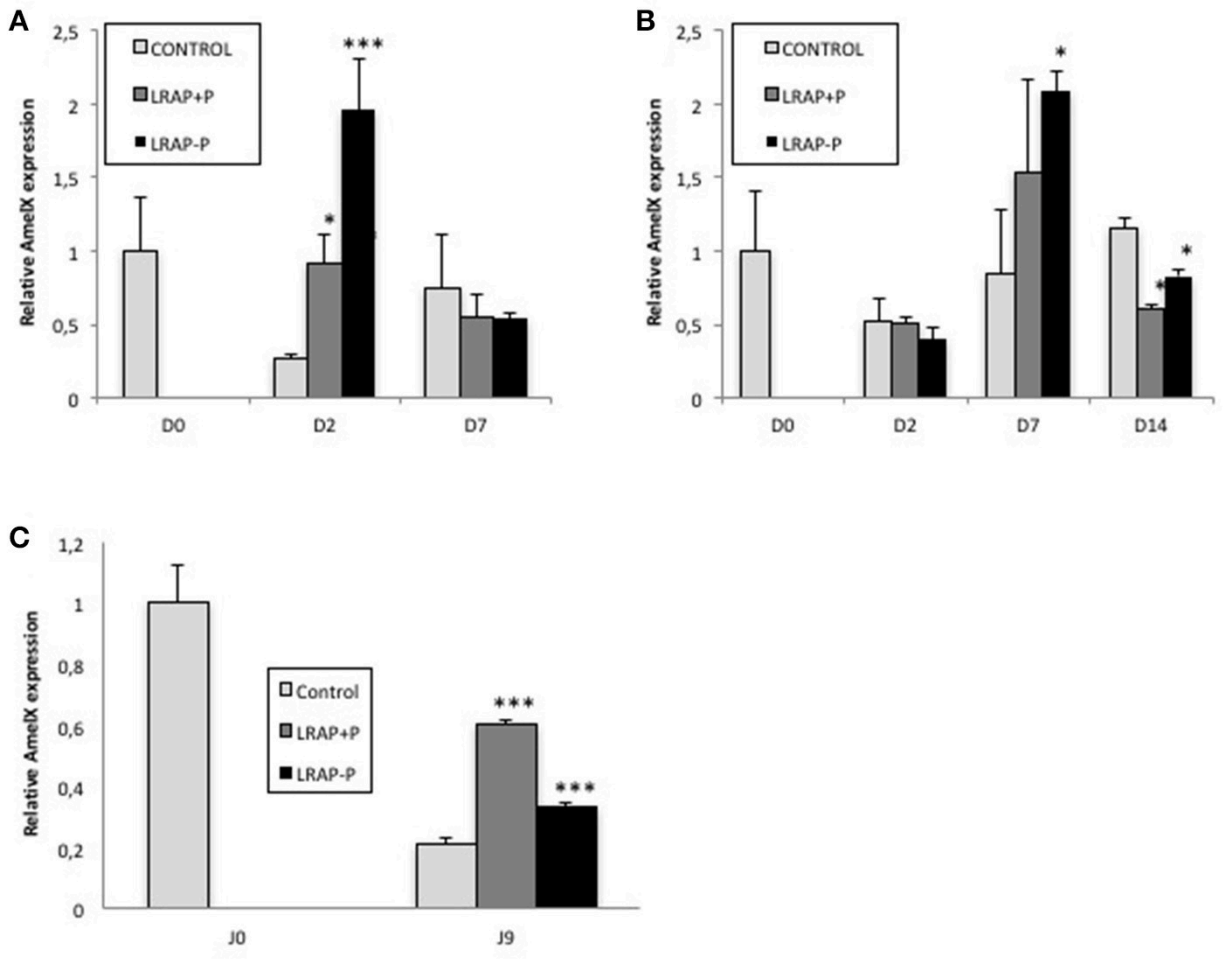
Kinetic of expression of amelogenin gene in LS8 and ALC cells and in the cultured first molar germs. **(A,B)** *Amelx* quantitative PCR analyses for the LS8 **(A)** and ALC **(B)** cell lines at selected time-points. *Amelx* expression was normalized to GAPDH and Actin for each time-point and cell line. Average expression levels and standard deviation error were calculated from 3 different qPCR experiments (each run in triplicate, *n* = 3). At D2, both peptides induced a statistically significant increase in amelogenin transcripts relative to the control in the LS8 [*p < 0.05 for LRAP(+P) and ^***^p < 10^−4^ for LRAP(–P)]. At D7, both peptides induced a similar increase for the ALC, statistically significant for LRAP(–P) (*p < 0.05) relative to the control. **(C)** *Amelx* transcripts levels were compared between D0 (PND1 germ) and cultured D9 germs. Inhibition of *Amelx* expression was observed in all conditions relative to the D0 germ. LRAP(+P) and LRAP(–P) treatment induced a statistically significant (*p < 0.05) increase in *Amelx* expression relative to the control.

Since the un-phosphorylated form of LRAP had been previously shown to impact *Amelx* gene transcription (Iacob and Veis, 2008), the relative effect of both peptides on *Amelx* expression by the cells was evaluated by qPCR. We observed that both forms of LRAP induced an early (D2) statistically significant up-regulation of *Amelx* transcription in the LS8 cells although more pronounced with LRAP(-P) (Figure 3A). In ALC, a similar up-regulation (D7) in *Amelx* transcription was observed with the two peptides, although only statistically significant with LRAP(–P) (Figure 3B). This up-regulation was however delayed as compared to LS8 cells in agreement with the mineralization kinetics (Figures 3A,B).

Therefore, both peptides affected amelogenin expression and presented an effect on crystal organization.

### Effect of LRAP peptides on germ mineralization

**Figure 4:**
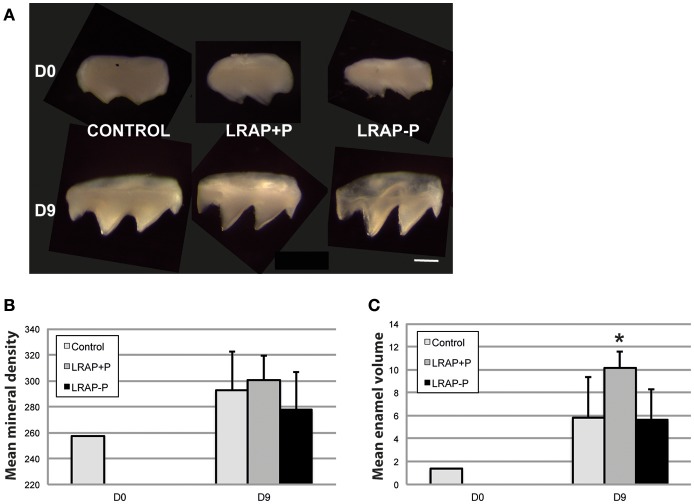
Macroscopic views and mineral density and enamel volume of first molar germs cultured in the absence and presence of added peptide. **(A)** Photographs of first molar germs at D0 and after 9 days of culture in the absence (CONTROL) or presence of LRAP(+P) and LRAP(–P). Scale bar 500 μm. Mean mineral density **(B)** and enamel volume **(C)** were calculated from Micro-CT scans of first molar germs at D0 and after 9 days of culture. Addition of LRAP(+P) peptide lead to a statistically significant increase (^*^p < 0.05) in enamel volume relative to the D9 control, no difference could be observed between LRAP(–P) treated and control samples. All samples presented an increased mineral density **(B)** and enamel volume **(C)** at D9 relative to D0 confirming tooth germ growth and maturation.

To determine the effect of LRAP phosphorylation on mineral formation, in a more integrated biological context, we tested the peptide effect on PND1 molar tooth germs cultured *ex vivo* over a 9 day period (Bègue-Kirn et al., 1992; Tompkins et al., 2005). Growth of the first molar germs was observed in all conditions upon 9 days of *ex vivo* culture on semi-solid medium (Figure 4A). Micro-CT imaging allowed quantifying germ mineralization in all samples, as well as determining the mineral density (enamel and dentin combined) and the enamel volume (Figures 4B,C). An increase in mineral density was detected in all cultured samples, i.e., treated and untreated, as compared to uncultured D0 germs confirming the germ growth in culture (Figure 4B). Peptide treatment did not appear to impact this value. In contrast, germ culture in the presence of LRAP(+P) led to an increase in enamel volume (>50%) as compared to untreated germs, in contrast to LRAP(–P) treatment which did not (Figure 4C).

**Figure 5:**
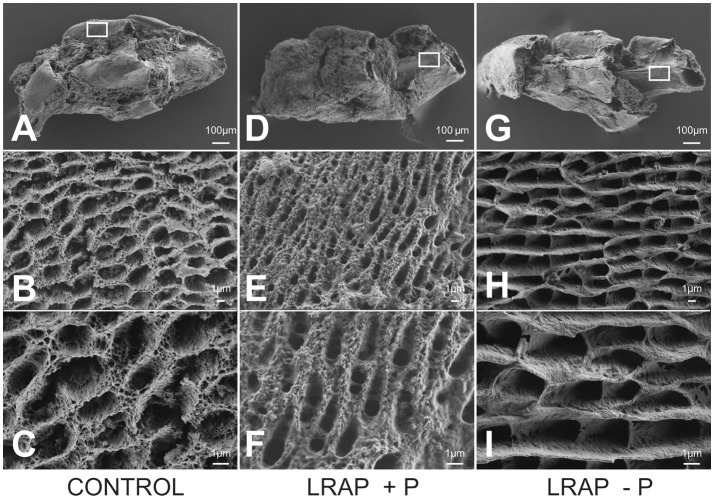
FE-SEM analyses of D9 first molar germs cultured in the absence and presence of added peptide **(A–C)** CONTROL, **(D–F)** LRAP(+P), and **(G–I)** LRAP(–P). Ameloblast pits and mineral organization were clearly observed for all three samples, confirming enamel growth in culture. Ameloblast pits appeared smaller and more spaced in molar germs treated with LRAP(+P) **(E,F)** relative to the control **(B,C)** and slightly wider in molar germs treated with LRAP(–P) **(H,I)** relative to the control.

The mineral formed in the PND1 molar tooth germs was further characterized by FE-SEM (Figure 5). Ameloblast pits typical of immature enamel were observed in all samples, confirming enamel formation in the culture process (Figures 5A,D,G). LRAP(+P) treated germs displayed smaller and more spaced pits (Figures 5E,F) than untreated controls (Figures 5B,C), while those of LRAP(–P) germs appeared slightly wider (Figures 5H,I) than the LRAP+P-treated or control germs. These observations suggested an increased mineralization process in the presence of LRAP(+P) confirming the micro-CT analysis.

The effect of the LRAP peptides on *Amelx* transcription was evaluated in the PND1 molar germs cultures. A statistically significant up-regulation of *Amelx* transcription was observed with both LRAP(+P) and LRAP(–P) treatment relative to untreated germs, with a stronger effect of LRAP(+P) than LRAP(–P) (respectively 3- vs. 1.5-fold relative to the control) (Figure 3C).

## Discussion

This study shows a differential effect of the LRAP peptide on enamel formation, depending on its phosphorylation status in *in vitro* and *ex vivo* culture models. Mature enamel, in contrast to other mineralized tissues like bone or dentin, cannot be repaired. When the tooth erupts in the oral cavity, ameloblasts are degraded and, consequently, the enamel cannot be re-grown or regenerated. The search for molecules able to restore enamel defects is therefore ongoing. In this context, the LRAP peptide has proven of interest thanks to its signaling properties as well as its apparent effect on crystal growth and structure (Shaw et al., 2004; Beniash et al., 2009; Le Norcy et al., 2011a,b; Wiedemann-Bidlack et al., 2011; Moradian-Oldak, 2012; Kwak et al., 2016, 2017).

In the present study, we first show that both forms of peptide have a differential effect on the mineral formed by ameloblast-like cells in culture. Untreated LS8 cells, a model for secretory stage ameloblast, thus actively expressing *Amelx, Ambn*, and *Enam*, and *Mmp20* mRNAs (Sarkar et al., 2014), synthesize crystals with a very similar structure to those observed in secretory stage tooth enamel. LRAP(-P) treatment potentiates the crystal lengthening and bundle formation whereas LRAP (+P) has little effect on the general crystal shape. Therefore, in the LS8 model, despite the up-regulation of *Amelx* expression promoted by both forms of the peptide, the structure of the mineral appears mainly affected by the LRAP(–P) form, likely through a direct action of the peptide on HAP crystals as previously observed in precipitation experiments (Le Norcy et al., 2011b).

In the ALC cell line, a model for maturation stage ameloblast, characteristically expressing *Amelx, Odam*, and *Klk4* transcripts, treatment by both peptides affects crystal formation, favoring bundle formation, as in LS8 cells. This effect is, however, again most evident with LRAP(–P). Remarkably, when measuring the length to width ratio, no significant difference is found between the crystals formed by control or LRAP-treated cells which might be related to the fact that this maturation stage is characterized by a crystal growth and no longer elongation (Sarkar et al., 2014). The fact that both peptides stimulate *Amelx* expression in the ALC cell, where it is usually low, could explain the change in crystal morphology and organization through guidance by the potentially newly induced amelogenin protein. The observed LRAP(–P) action could then result from a direct effect of the peptide on crystal shape as observed in LS8 cells and in precipitation experiments (Le Norcy et al., 2011a,b) and recently on acid etched enamel surfaces of human teeth (Kwak et al., 2017).

Molar germs can be cultured *ex vivo* and were shown to develop well-organized layers of polarized ameloblasts and odontoblasts (Tompkins and Veis, 2002). Micro-CT and FE-SEM analyses of the mineral formed by PND1 molar germs after a 9-day culture revealed an increase in enamel volume with LRAP(+P) while it was not increased by LRAP(–P) treatment. Rescue experiments with recombinant plasmid encoding LRAP in amelogenin KO mice, have shown that LRAP contributes to final enamel thickness and prism organization (Gibson et al., 2011; Xia et al., 2016). It can be speculated from our data, that in these mice LRAP is present under its phosphorylated form.

Altogether our results obtained in cell and germ cultures claimed for a differential effect of the phosphorylated and non-phosphorylated LRAP on crystal formation. It is not clear however at this point why LRAP(–P) did not significantly impact the mineral volume in the germ culture while it did so in the cell lines. This observation might be related to the 3D vs. 2D cell organization in both system and to the homogeneous differentiation stage present in the cell cultures as compared to the cultured germs where secretory and mature ameloblasts co-exist.

Our results on the peptide action on ameloblast cell-like culture, strongly suggest that peptide phosphorylation is not essential to achieve an impact on amelogenin gene expression, and likely on differentiation, since LRAP(+P) as well as LRAP(−P) were both able to stimulate amelogenin transcripts in the LS8 and ALC cells. This stimulation is restricted in a time frame since for both cell lines and peptides, it is followed by a decrease in amelogenin expression in agreement with what is observed during the process of differentiation of tooth ameloblasts. Our results with LRAP(–P) are concordant with previous studies showing its action on ameloblastic differentiation (Tompkins and Veis, 2002; Tompkins et al., 2005; Ravindranath et al., 2007). Notably, the two cell lines reacted to the peptide treatment with a different kinetics. The LS8 cell line responded very quickly to LRAP treatment (48 h) by increasing the number of *Amelx* transcripts whereas the ALC cell response was delayed (7 days). This variation in response kinetics is likely linked to the different stage of ameloblastic differentiation mimicked by these cell lines. Amelogenin secretion is very active during the secretory stage (Aoba et al., 1987), but then drops as the cells mature. In the LS8 cells, the peptides appear to potentiate the already active expression of amelogenin and this process appears direct as shown previously for LRAP(–P) (Iacob and Veis, 2008) while in the ALC cells they likely act through indirect more complex processes.

Understanding the potential complementary action of LRAP(+P) and LRAP(–P) on cell mineralization and metabolism is a next step in our analysis. This may further lead to the establishment of differential treatments by selected peptide(s) according to the tooth developmental stage.

The present data obtained in biological models parallel those recently described *in vitro* claiming that LRAP(–P) is involved in the modulation of crystal maturation (length, width) (Shafiei et al., 2015; Kwak et al., 2017). Therefore, its topical application can be envisioned for a future repair of enamel lesions. Furthermore, our *ex vivo* observations on LRAP(+P) correlate with the recent *in vitro* findings by Yamazaki and colleagues on the native phosphylated amelogenins during the early stages of enamel formation (Yamazaki et al., 2017). Unraveling the signaling pathways underlying this action is therefore mandatory for a potential use of this peptide as early treatment of inborn disorders of enamel.

## Author contributions

ELN: Designed the setup of experiments, performed experiments, and drafted the manuscript; JL: Performed experiments. JS: Performed experiments. GR: Participated in result analysis. CC: Participated in drafting the manuscript. AP: Designed the setup of experiments, and drafted the manuscript.

### Conflict of interest statement

The authors declare that the research was conducted in the absence of any commercial or financial relationships that could be construed as a potential conflict of interest. The reviewer CC and handling Editor declared their shared affiliation.
