# Supplementary figures and images for: Phosphorylated and Non-phosphorylated Leucine Rich Amelogenin Peptide Differentially Affect Ameloblast Mineralization
Source: Front Physiol. 2018 Feb 8;9:55. doi: 10.3389/fphys.2018.00055 (PMC5809816; doi:10.3389/fphys.2018.00055)

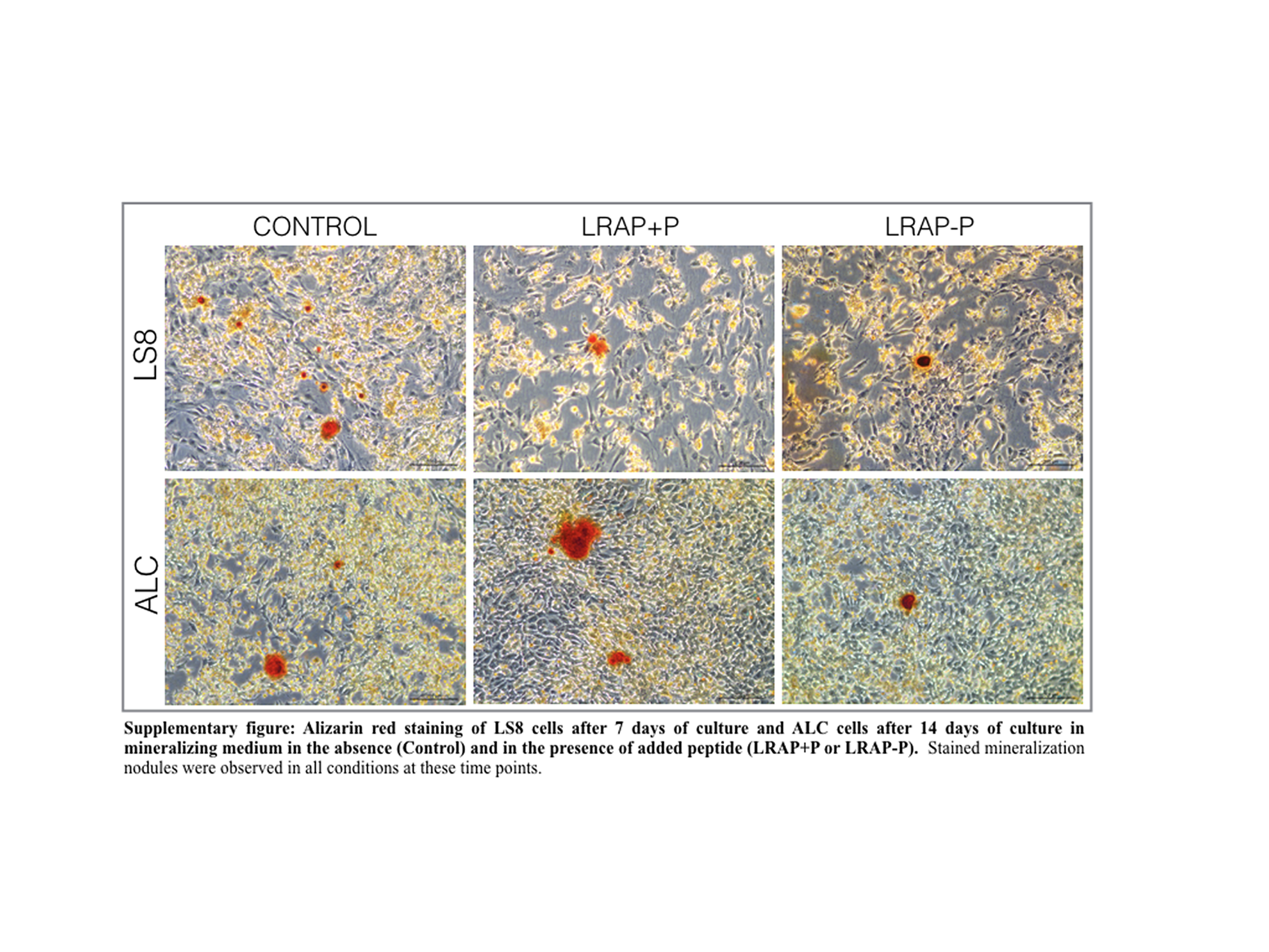

Supplement: Supplementary file 1 [file Image1.TIFF]
